# Improving the Commercial Value of the ‘Calçot’ (*Allium cepa* L.) Landrace: Influence of Genetic and Environmental Factors in Chemical Composition and Sensory Attributes

**DOI:** 10.3389/fpls.2018.01465

**Published:** 2018-10-04

**Authors:** Silvia Sans, Joan Casals, Joan Simó

**Affiliations:** ^1^Miquel Agustí Foundation, Castelldefels, Spain; ^2^Department of Agri-Food Engineering and Biotechnology, Universitat Politecnica de Catalunya, Castelldefels, Spain

**Keywords:** landrace, ‘calçot’, onion, chemical composition, sensory attributes, environmental influence

## Abstract

Landraces are considered valuable for their close ties to local cultures, adaptation to low inputs, and quality. ‘Calçots’ are the immature floral stems of second-year sprouts of onions from the ‘Blanca Tardana de Lleida’ landrace. ‘Calçots’ grown in their traditional area of cultivation have been awarded Protected Geographic Indication (PGI) ‘Calçot de Valls’ from the European Union. Despite annual sales of about €15 million, ‘calçot’ germplasm and cultivation methods are under-researched. This study aimed to estimate the influence of genetic and environmental factors in the chemical and sensory characteristics of ‘calçots’ to enable strategies to improve their commercial value to be devised. To this end, we tested the landrace and three new, more productive varieties derived from the landrace in experiments conducted over two seasons in six locations (within and outside the PGI zone), using two planting dates and two harvesting times. The results point to a major environmental influence in the quality of ‘calçots.’ The analysis of variance found all factors related with environmental influence were significant in most chemical traits considered (dry matter content, soluble solids content, pH, titratable acidity, and ash content), while the variety factor was significant only for titratable acidity. In sensory analyses, the variety factor and all the environmental factors had significant effects in all sensory traits recorded (sweetness, fiber perception, and off-flavors). In both chemical and sensory traits, most significant interactions involved the environmental factors. The negative correlation found between sweetness and fiber perception and off-flavors suggests that additional selection can bring ‘calçots’ closer to the sensory ideotype. Although clearly more productive, the new ‘calçot’ varieties maintain the chemical composition and sensory value of the landrace. Thus, fine-tuning the cultivation and/or breeding of the landrace for both yield and quality seem viable approaches to obtaining better commercial products.

## Introduction

Landraces are important resources in agriculture for their adaptation to particular environments and low inputs, their close ties to local cultures, and their tolerance and resistance to biotic and abiotic stresses (Newton et al., 2010; Casañas et al., 2017). Landraces renowned for their high sensory quality have maintained a commercial role in specialist production for niche markets (Villa et al., 2005). Extensive discussions have sought to define the concept of landrace, and many authors have associated landraces with a lack of formal genetic improvement (Zeven, 1998; Villa et al., 2005). Recently, Casañas et al. (2017) proposed to define landraces as cultivated varieties that have evolved and may continue to evolve through the use of conventional or modern breeding techniques in traditional or new agricultural environments within a defined ecogeographical area under the influence of the local human culture.

To recognize the added value of high quality local products and enhance rural development, the European Union promotes three types of food quality labels: Protected Designation of Origin (PDO), Protected Geographical Indication (PGI), and Traditional Speciality Guaranteed (TSG). When applied to vegetables, PDO and PGI are closely tied to the geographical area of production and to the genotype-by-environment (GxE) interaction, which usually involves landraces. Thus, these designations simultaneously promote rural development and the *in situ* conservation of landraces (Smale et al., 2004). These food quality labels also help consumers identify products and crop varieties associated with cultural or biological heritage within a limited geographical area (Veteläinen et al., 2009). Products under food quality labels have singular organoleptic and/or nutritional traits derived from historically selected GxE interactions. To enhance these traits, some quality labels are incorporating descriptive sensory analysis through trained panels for quality control to ensure the sensory characteristics of their products (Pérez-Elortondo et al., 2018).

‘Calçots’ are the immature floral stems of second-year onion (*Allium cepa* L.) resprouts, mainly from the long-day ‘Blanca Tardana de Lleida’ (BTL) landrace, typically roasted on a hot open fire in Catalonia (Northeast Spain). The BTL landrace is characterized by late development, white skin and flesh, and the production of between 1 and more than 25 resprouts (‘calçots’) per onion. The European Union has designated the PGI ‘Calçot de Valls’ for ‘calçots’ from the BTL landrace of onions cultivated in the traditional area of cultivation (EC No 905/2002, 2002). There are no official economic data about ‘calçots,’ but it is estimated that the current market volume is about €15 million. Moreover, agro-tourism related with ‘calçots’ boosts the regional economy and has increased interest in demand for ‘calçots’ worldwide. The recent surge in commercialization has made farmers more interested in improving the quality and homogeneity of their product. To date, the regulating board of the PGI ‘Calçot de Valls’ has focused quality control on parameters related to external appearance (length and width of the edible part), but producers trading under the label aim to expand quality control to include quality-related parameters.

The agronomic performance of ‘calçots’ has been studied, and some tools have been developed to facilitate breeding for yield (Simó et al., 2013). As a result, two new more productive varieties have been obtained: Roquerola, which provides 320% more commercial-sized ‘calçots’ in early harvests, and Montferri, which provides 116% more ‘calçots’ in late harvests compared with the base population (Simó et al., 2012a). In parallel, a sensory ideotype has been elaborated; the ideal ‘calçot’ should have a high level of sweetness, low fiber perception, and no off-flavors (Simó et al., 2012b). In recent years, farmers of PGI ‘Calçot de Valls’ have relied heavily on the new varieties, but some historical populations are still cultivated. No breeding programs have been developed to improve the sensory quality of ‘calçot’ crop.

The chemical and nutritional composition, as well as the sensory profile of the plants, is determined by genetic and environmental factors and their interactions (Allard, 1999). In PDO or PGI products, the specific quality profile is conferred by the interaction between the genotype (i.e., landraces) and the environment (i.e., the historical area of production) (Romero del Castillo et al., 2008), and research programs should identify the genetic and environmental factors underlying these traits. For this reason, it is important to conduct studies that increase our understanding of the factors influencing quality.

As a first step toward expanding the attributes specified in the PGI ‘Calçot de Valls’ to include sensory traits, this study aimed to estimate the influence of genetic and environmental factors in some key chemical and sensory traits of ‘calçots.’

## Materials and Methods

### Experimental Design

**FIGURE 1:**
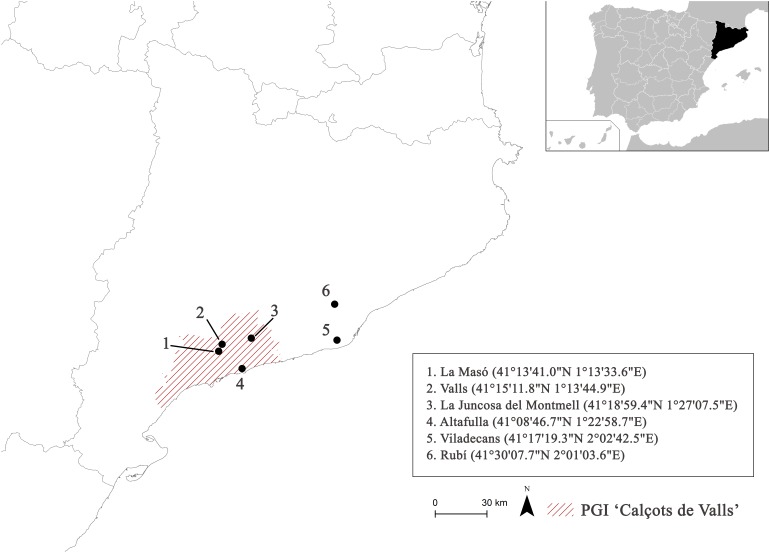
Map of the experimental field locations.

Field experiments were conducted in two consecutive seasons (2014–2015 and 2015–2016) at six different locations with different pedo-climatic conditions in Catalonia (Northeast Spain) that represent standard ‘calçot’ production areas (**Figure 1**). Four experimental fields were located within the geographical area designated by the PGI, while the two others were outside the designated area.

Four different varieties of BTL were evaluated: the original population, which has not undergone any formal scientific breeding processes, the improved varieties Roquerola and Montferri, derived from the historical landrace by scientific breeding (Simó et al., 2012a), and a new experimental variety with higher yields, also derived from the historical landrace by scientific breeding. To avoid the effect of the first growing cycle conditions on ‘calçots’ cultivation, bulb onions used in the experiments were produced in the same field (Simó et al., 2013). In both experimental seasons, bulb onions were replanted at a density of 32,000 plants per hectare, using a planting pattern of 0.3 × 0.75 m, at two different times, in mid-August (early planting) and in late September (late planting). ‘Calçots’ were harvested at two different times, in December (early harvest) and in February (late harvest). The experimental design was three randomized blocks, with 50 plants per plot. Each experimental field was managed by farmers using their own customary traditional cultivation techniques. The fertilization, irrigation, weed control and pest management were also managed using the farmer cultivation practices.

### Soil Characteristics and Climate Data

Before planting, soil analyses were performed for each location. A hollow cylindrical corer with an internal diameter of 7 cm was used to collect seven 25-cm deep subsamples along a zigzag path from each experimental field. Subsamples were mixed to obtain homogeneous samples of about 1000 g for each site. The analyses were performed to evaluate the following soil properties: pH, electrical conductivity, percentage of organic matter, percentage of calcium carbonate equivalent, content of N, P, K, Ca, Mg, and Na, USDA textural class, and cation exchange capacity.

Climatic data (mean maximum and minimum monthly temperatures) were obtained from meteorological stations located near the experimental fields (**Table 2**). Rainfall was not considered because there was no unusual episode of rain and all the fields were cultivated under irrigation.

### Sample Preparation

For each treatment (combination of variety, year, location, planting date, and harvest date) we collected three different samples. Each sample comprised a set of 80 commercial ‘calçots’ (PGI regulations define commercial ‘calçots’ as having a compact white edible base measuring 15–25 cm in length and 1.7–2.5 cm in diameter 5 cm from the root). ‘Calçot’ samples were prepared as described by Simó et al. (2012b). Leaves were cut 4 cm above the ligule, and roots were removed. Then, ‘calçots’ were rinsed with tap water to remove adhered soil and roasted at 270°C for 18 min in a convection oven (SALVA Kwik-co). After cooking, the two most external leaves were removed and the edible lower white part of each ‘calçot’ was cut. All ‘calçots’ in each sample were triturated with a mixer (Taurus BAPI 850). Pureed samples were frozen with liquid nitrogen and stored at −20°C until their chemical and sensory analyses.

### Chemical Analysis

Soluble solids content was directly determined in the puree with a hand refractometer (Erma, Japan) and expressed as ^o^Brix. To analyze titratable acidity, 10 g of puree was mixed with 50 mL of distilled water, initial pH was recorded, and then the mixture was titrated with 0.1 M sodium hydroxide (NaOH) to pH 8.1; titratable acidity was expressed as g/100 g of malic acid. To determine dry matter, 30 g of puree was dried to a constant weight for 72 h at 60°C; dry matter was expressed as g/100 g of fresh matter. To determine ash content, we used AOAC method 923.03 (AOAC, 2005): dried samples were ground to an average particle size < 0.4 mm to obtain flour; then, 1 g of flour was burned in a muffle at 450°C for 4 h, cooled to room temperature in a desiccator, and finally weighed. Ash was expressed as g/100 g of dry matter. All chemical analyses were carried out in triplicate.

### Sensory Analysis

Descriptive sensory analysis requires trained panels, and these panels can work with a limited number of samples. This limitation precluded panel analysis of the nearly 200 samples generated along the experiment; therefore, sensory analysis consisted only of a preliminary survey using selected samples from the second year. Thus, the panel tested a subset of 32 samples representing the early and late harvests of the 4 varieties in 4 locations (La Masó, La Juncosa del Montmell, Valls, and Viladecans).

Sensory analysis was carried out as described by Simó et al. (2012b). Each of the 8 trained panelists evaluated the samples of puree in duplicate in a total of 13 sessions. Sensory attributes (sweetness, fiber perception, and off-flavors) were measured on semi-structured visual scales labeled from 0 to 10. All tests were carried out in a room designed for sensory tests that fulfilled the standards set out by the International Organization for Standardization (ISO 8589, 2007).

### Statistical Analysis

Data were analyzed with R statistic software (R Core Team, 2017). PLS_Toolbox v.8.21 software (Eigenvector Research Inc., Wenatchee, WA, United States) was used for principal components analysis (PCA).

Each chemical and sensory trait was studied by ANOVA to detect statistical significance, according to the following linear models:

For chemical attributes,

(1) $$\begin{array}{c} X_{\mathrm{ijklm}}=\mu +v_{i}+l_{j}+s_{k}+p_{l}+h_{m}+v_{i}l_{j}+v_{i}s_{k}+v_{i}p_{l}+v_{i}h_{m}\\ +l_{j}s_{k}+l_{j}p_{l}+l_{j}h_{m}+s_{k}p_{l}+s_{k}h_{m}+p_{1}h_{m}+\varepsilon_{\mathrm{ijklm}}, \end{array}$$

and for sensory attributes,

(2) $$\begin{array}{c} X_{\mathrm{ijkl}}=\mu +v_{i}+l_{j}+h_{k}+t_{l}+v_{i}l_{j}+v_{i}h_{k}+v_{i}t_{l}+l_{j}h_{k}\\ +l_{i}t_{l}+h_{k}t_{l}+\varepsilon_{\mathrm{ijkl}}, \end{array}$$

where v, l, s, p, h, and t are the factors variety, location, season, planting date, harvesting time, and trained panelist, respectively. All factors were considered fixed. Means of significant factors were compared by calculating the least significant difference (LSD) (*P* < 0.05). We used Pearson’s correlation coefficient and regression to study the relations among the traits.

## Results

### Environmental Description

Soil texture was classified as loamy in all the experimental fields. The results of the remaining parameters studied (**Table 1**) showed that in general all the locations were calcareous, presented between basics and slightly alkaline soils and low concentration of organic matter, especially Viladecans during the first season (0.71%). In no case electrical conductivity was a limiting factor for ‘calçot’ cultivation.

**Table 1:** Soil properties at the six locations in the two seasons.

Trait	Season	Altafulla	La Juncosa	La Masó	Rubí	Valls	Viladecans
Soil pH	14–15	8.54	8.47	8.38	8.43	8.27	8.57
	15–16	8.61	8.40	8.37	8.48	8.28	8.43
Electrical conductivity 25°C (dS m^−1^)	14–15	0.232	0.152	0.194	0.19	0.194	0.265
	15–16	0.212	0.174	0.223	0.18	0.22	0.275
Organic matter (%)	14–15	1.52	1.63	1.72	1.71	1.76	0.71
	15–16	1.12	1.30	1.34	1.28	1.3	1.10
Calcium carbonate equivalent (%)	14–15	36	38	39	23	52	24
	15–16	42	47	38	21	51	32
Nitrogen (mg N-NO3 kg^−1^)	14–15	40	18	32	36	78	22
	15–16	22	14	23	69	39	47
Phosphorus (mg kg^−1^)	14–15	20	21	27	18	3	9
	15–16	37	36	95	33	16	49
Potassium (mg kg^−1^)	14–15	230	590	374	334	292	547
	15–16	155	626	340	205	261	293
Calcium (mg kg^−1^)	14–15	6,529	6,979	6,456	6,555	7,066	7,150
	15–16	5,989	6,828	6,032	6,066	6,689	6,863
Magnesium (mg kg^−1^)	14–15	593	705	379	280	374	329
	15–16	499	531	344	300	358	337
Sodium (mg kg^−1^)	14–15	89	25	43	101	26	142
	15–16	157	17	35	41	44	104
Cation exchange capacity (cmol kg−1)	14–15	7.1	14.2	8.0	11.3	9.8	6.8
	15–16	6.3	15.7	7.5	11.8	10.4	9.9

Temperatures at all locations were characteristic of the mild Mediterranean climate, with marked differences over the months (**Table 2**). In general, the 2014-2015 season was warmer during the autumn, but in winter the highest temperatures were recorded in the 2015–2016 season.

**Table 2:** Climatic data (°C) of the six locations in the two experimental seasons.

Location	Season	August		September		October		November		December		January		February	
		
		Mean min.	Mean max.	Mean min.	Mean max.	Mean min.	Mean max.	Mean min.	Mean max.	Mean min.	Mean max.	Mean min.	Mean max.	Mean min.	Mean max.
Altafulla	14–15	21.2	28.3	19.7	26.9	15.1	24.3	11.4	19.7	5.7	15.3	4.8	14.7	4.4	14.4
	15–16	20.9	29.3	17.2	25.3	13.7	22.2	9.5	19.3	8.2	16.7	7.5	16.2	6.8	17.0
La Juncosa	14–15	16.7	28.3	15.9	26.1	13.8	24.1	9.0	15.9	4.8	12.4	3.6	12.5	2.1	11.4
	15–16	17.0	29.6	13.5	24.2	10.9	20.4	8.5	18.1	7.1	15.6	5.3	13.4	5.2	14.1
La Masó	14–15	20.1	30.0	18.5	28.7	14.6	24.5	10.3	18.5	5.5	12.7	4.3	13.3	4.1	13.9
	15–16	20.4	31.1	16.6	25.6	12.5	21.6	9.1	17.7	7.1	15.6	6.3	15.5	6.4	16.1
Rubí	14–15	18.8	29.9	17.2	27.9	13.5	25.9	8.8	18.1	3.4	13.0	2.3	13.7	2.0	14.2
	15–16	18.6	31.4	15.1	26.2	11.7	22.6	7.0	19.4	5.0	16.4	5.1	15.8	4.8	17.2
Valls	14–15	17.6	28.1	16.4	26.7	12.4	24.3	8.3	17.7	3.5	13.0	2.2	12.9	2.0	12.7
	15–16	17.8	29.1	13.8	24.3	10.5	21.0	6.8	17.6	4.9	15.6	4.8	14.6	4.4	15.2
Viladecans	14–15	20.2	28.9	18.9	27.2	14.8	24.6	10.4	19.2	5.5	14.6	4.3	14.7	4.0	14.2
	15–16	20.2	30.6	16.8	26.1	13.2	22.6	8.9	19.6	7.2	17.1	6.4	16.4	5.7	17.3

*Mean min.: mean of monthly minimum temperatures; Mean max.: mean of monthly maximum temperatures.*

**FIGURE 2:**
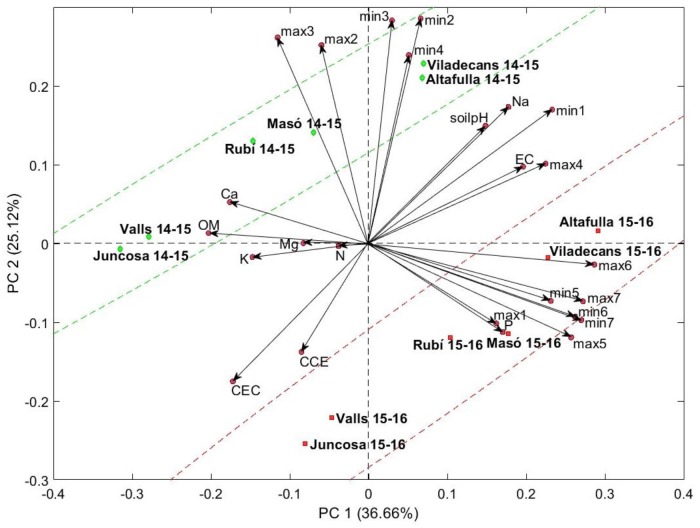
Biplot of the location × season in the plane determined by the first two axes of the PCA considering the soil characteristics and climatic conditions. The angle of the vector with the axes indicates the correlation between the principal component and the original variable, and its length is proportional to the variability in the original variable explained by each principal component. The percentages between parentheses refer to the variation explained by each principal component. soilpH, pH of the soil; EC, electrical conductivity; OM, organic matter; CCE, calcium carbonate equivalent; N, nitrogen; P, phosphorus; K, potassium; Ca, calcium; Mg, magnesium; Na, sodium; CEC, cation exchange capacity; min, mean of the minimum temperatures for each month; max, mean of the maximum temperatures for each month; 1, 2, 3, 4, 5, 6, and 7 indicate August, October, November, December, January, and February, respectively.

Principal components analysis was performed on environmental characteristics (soil and climate data). The first three principal components, accounting for 74.9% of the total variance, revealed strong differences between seasons. The first component (PC1, 36.7%) was primarily correlated positively by mean minimum temperatures in December, January, and February; mean maximum temperatures in November, December, January, and February; sodium content; and electrical conductivity. Three factors correlated negatively with the first component: calcium content, cation exchange capacity, and organic matter. The traits that correlated positively most strongly with the second component (PC2, 25.1%) were mean minimum temperatures in August, September, October, and November; mean maximum temperatures in September and October; and soil pH. Two factors correlated negatively with the second component: cation exchange capacity and calcium carbonate equivalent. PCA revealed similarities among some locations, grouping them in pairs: Altafulla and Viladecans, La Masó and Rubí, and Valls and La Juncosa. However, the effect of the season seemed stronger since there was a clear displacement of all the locations between the two seasons studied, due to higher content of P and higher temperatures during the winter months (**Figure 2**).

### Chemical Attributes

**FIGURE 3:**
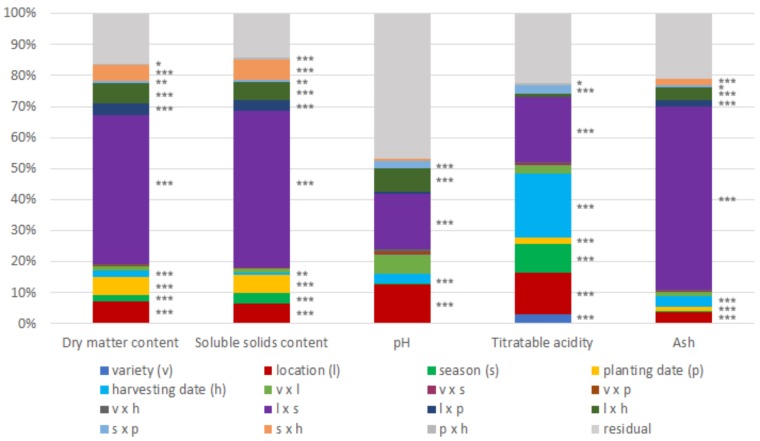
Percentage of variance due to the factors considered in the ANOVA (*n* = 576) and their interactions in the chemical parameters studied. Significance codes of the factors considered in the ANOVA: ^∗∗∗^*P* < 0.001, ^∗∗^*P* < 0.01, ^∗^*P* < 0.05.

The analysis of variance showed a major environmental influence in chemical traits of ‘calçots.’ All factors related with environmental influence (location, planting date, harvesting time, and season) were significant (*P* < 0.05) for all the chemical traits considered, except the factors season and planting date for pH and the factor season for ash content. By contrast, the factor variety was significant only for the attribute titratable acidity. The only significant interactions were between factors related with environmental influence, being the interaction location × season the most important (**Figure 3**).

The greatest differences were found between locations (**Table 3**). Differences between locations ranged from 1.9% for pH to 34.6% for titratable acidity. Valls, La Juncosa, and Altafulla had the highest values for dry matter and soluble solids content, while Rubí had the lowest mean values for titratable acidity and ash content. As mentioned above, the only chemical trait that was significantly different between varieties was titratable acidity, which was highest in the “traditional” landrace and lowest in the new “experimental” variety (**Table 3**). The amount of dry matter and soluble solids content were higher in the first season (2014–2015), and titratable acidity was higher in the second season (2015–2016). On average, ‘calçots’ planted early (in August) presented higher values of dry matter and soluble solids contents and lower values of titratable acidity and ash content. ‘Calçots’ from the early harvest presented the highest values for all the chemical parameters analyzed except pH.

**Table 3:** Means and standard deviations of chemical attributes between varieties, locations, seasons, planting dates, and harvesting times.

Factor	Dry matter content (g/100 g f.m.)	Soluble solids content (^*o*^Brix)	pH	Titratable acidity (g malic acid /100 g f.m.)	Ash (g/100 g d.m.)
**Variety**					
Roquerola	15.946 ± 0.249a	12.7 ± 0.2a	6.10 ± 0.01a	0.127 ± 0.004b	5.129 ± 0.088a
Montferri	15.898 ± 0.239a	12.8 ± 0.2a	6.09 ± 0.02a	0.124 ± 0.003bc	5.155 ± 0.109a
Traditional	16.033 ± 0.224a	12.8 ± 0.2a	6.11 ± 0.01a	0.133 ± 0.004a	5.147 ± 0.100a
Experimental	15.803 ± 0.238a	12.6 ± 0.2a	6.09 ± 0.02a	0.121 ± 0.003c	5.152 ± 0.089a
% variation	–	–	–	9.9%	–
**Location**					
La Masó	15.575 ± 0.248c	12.4 ± 0.2c	6.09 ± 0.02bc	0.135 ± 0.005b	5.261 ± 0.072ab
Valls	16.609 ± 0.352a	13.4 ± 0.3a	6.04 ± 0.01d	0.144 ± 0.005a	4.995 ± 0.092cd
La Juncosa	16.066 ± 0.166b	12.8 ± 0.1b	6.07 ± 0.01cd	0.120 ± 0.002c	5.327 ± 0.135a
Altafulla	16.320 ± 0.159ab	13.1 ± 0.1ab	6.15 ± 0.02a	0.125 ± 0.004c	5.157 ± 0.053abc
Viladecans	15.383 ± 0.330c	12.2 ± 0.3c	6.11 ± 0.01b	0.123 ± 0.004c	5.148 ± 0.138bc
Rubí	15.324 ± 0.355c	12.3 ± 0.3c	6.10 ± 0.02bc	0.107 ± 0.004d	4.835 ± 0.105d
% variation	8.4%	9.5%	1.9%	34.6%	10.2%
**Season**					
14–15	16.226 ± 0.159a	13.1 ± 0.1a	6.10 ± 0.01a	0.118 ± 0.002b	5.088 ± 0.075a
15–16	15.622 ± 0.171b	12.4 ± 0.2b	6.09 ± 0.01a	0.134 ± 0.002a	5.202 ± 0.060a
% variation	3.8%	5.6%	–	13.6%	–
**Planting date**					
August	16.153 ± 0.143a	13.0 ± 0.1a	6.09 ± 0.01a	0.125 ± 0.002b	5.089 ± 0.053b
September	15.291 ± 0.185b	12.1 ± 0.2b	6.11 ± 0.01a	0.131 ± 0.004a	5.301 ± 0.103a
% variation	5.6%	7.1%	–	4.8%	4.2%
**Harvesting time**					
Early	16.419 ± 0.220a	13.0 ± 0.2a	6.07 ± 0.01b	0.143 ± 0.002a	5.290 ± 0.075a
Late	15.634 ± 0.131b	12.6 ± 0.1b	6.11 ± 0.01a	0.117 ± 0.002b	5.062 ± 0.061b
% variation	5.0%	3.8%	0.6%	22.2%	4.5%

*Within columns and for each factor, means followed by the same letter were not significant different at P ≤ 0.05 (least significant difference test). f.m., fresh matter; d.m., dry matter.*

### Relationships Between Environmental Variables and Chemical Composition of ‘Calçots’

Direct correlations were calculated between means of location × season of chemical parameters and environmental characteristics. Correlations were not robust, due to the complexity of the environmental factors. The only significant correlations (*P* < 0.05) were between soil pH and soil sodium content with the chemical trait pH of ‘calçots’ (*R* = 0.8 and *R* = 0.61, respectively) and between calcium carbonate equivalent and titratable acidity (*R* = 0.59).

**FIGURE 4:**
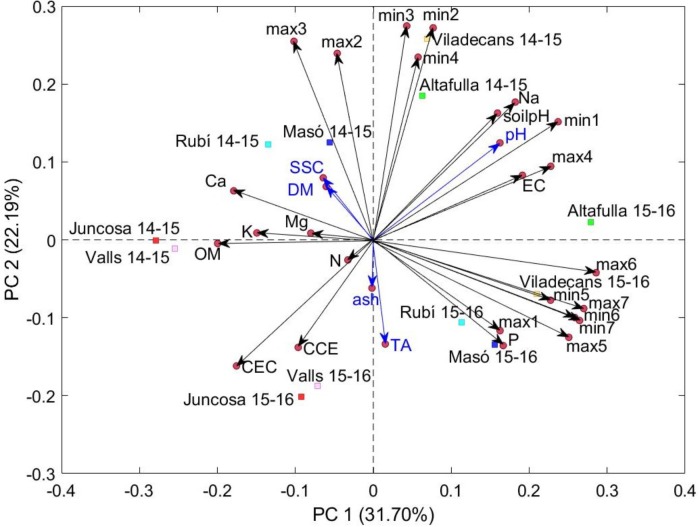
Biplot of location × season in the plane determined by the first two axes of the principal component analysis considering the environmental data and chemical traits. The angle of the vector with the axes indicates the correlation between the principal component and the original variable, and its length is proportional to the variability in the original variable explained by each principal component. The percentages between parentheses refer to the variation explained by each principal component. DM, dry matter content; SSC, soluble solids content; TA, titratable acidity; soilpH, pH of the soil; EC, electrical conductivity; OM, organic matter; CCE, calcium carbonate equivalent; N, nitrogen; P, phosphorus; K, potassium; Ca, calcium; Mg, magnesium; Na, sodium; CEC, cation exchange capacity; min, mean of the minimum temperatures for each month; max, mean of the maximum temperatures for each month; 1, 2, 3, 4, 5, 6, and 7 indicate August, October, November, December, January, and February, respectively.

Principal components analysis was applied using the means of chemical parameters in conjunction with environmental data (soil characteristics and climate data) of the 12 location × season combinations (**Figure 3**). The first three components explained 67.2% of the total variance, less than the PCA performed only with the environmental data (**Figure 2**). The two first components (PC1, 31.7%; PC2, 22.2%) were principally influenced by environmental characters (**Figure 4**) and were not notably different from the PCA that did not include the chemical parameters (**Figure 2**). The third component (PC3, 13.3 %) was strongly influenced by the chemical parameters dry matter and soluble solids content, with positive correlations, and ash content, with a negative correlation.

### Sensory Attributes

**FIGURE 5:**
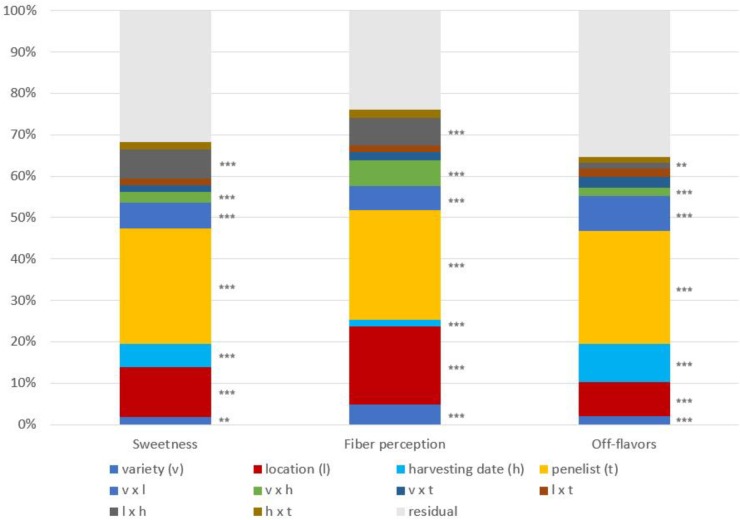
Percentage of variance due to the factors considered in the ANOVA (*n* = 96) and their interactions in the sensory attributes studied. Significance codes of the factors considered in the ANOVA: ^∗∗∗^*P* < 0.001, ^∗∗^*P* < 0.01, ^∗^*P* < 0.05.

All the main factors (variety, location, and harvesting time) were highly significant (*P* < 0.01) for all sensory traits, and the interactions between those factors were significant too. The factor panelist was also significant for the three sensory attributes considered, but none of the interactions that included the panelist factor were significant. In contrast to chemical parameters, we found significant differences between varieties; however, the factor variety explained low percentages of the variation and was not the most influential factor in any sensory trait (**Figure 5**).

On average, the differences between varieties for sweetness were very low. Montferri had higher values for fiber perception and was among the varieties with highest values for off-flavors. Among locations, La Juncosa had the highest values for sweetness and lowest for off-flavors; Valls was the location with the lowest values for fiber perception. On average, ‘calçots’ from the late harvest had a sensory profile more in line with the ideotype, being sweeter and less fibrous, with less off-flavors (**Table 4**).

**Table 4:** Means and standard deviations of sensory attributes between varieties, locations, and harvesting times.

Factor	Sweetness	Fiber	Off-flavors
**Variety**			
Roquerola	6.8 ± 0.1a	1.8 ± 0.1c	2.1 ± 0.2a
Montferri	6.3 ± 0.2b	2.9 ± 0.2a	2.2 ± 0.2a
Traditional	6.5a ± 0.2b	2.1 ± 0.2bc	2.1 ± 0.2a
Experimental	6.6 ± 0.2a	2.3 ± 0.2b	1.6 ± 0.2b
% variation	8.4%	57.9%	43.2%
**Location**			
La Masó	6.6 ± 0.2b	1.9 ± 0.1c	2.4 ± 0.2a
Valls	6.8 ± 0.2b	1.6 ± 0.1d	1.9 ± 0.2b
La Juncosa	7.2 ± 0.1a	2.2 ± 0.2b	1.2 ± 0.1c
Viladecans	5.8 ± 0.1c	3.6 ± 0.2a	2.5 ± 0.2a
% variation	23.0%	131.0%	111.6%
**Harvesting time**			
Early	6.2 ± 0.1b	2.5 ± 0.1a	2.5 ± 0.2a
Late	6.9 ± 0.1a	2.1 ± 0.1b	1.4 ± 0.1b
% variation	10.8%	23.0%	76.4%

*Season 2015–2016. Within columns and for each factor, means followed by the same letter were not significant different at *P* ≤ 0.05 (least significant difference test).*

### Correlations Among Chemical and Sensory Attributes

Overall, there were strong correlations between the chemical parameters dry matter, soluble solids, and ash content (**Table 5**). In contrast, pH and titratable acidity did not correlate with any of the other chemical parameters evaluated. Among the sensory attributes, we found negative correlations between sweetness and the other two (fiber, off-flavor). Analyzing the relationships between chemical and sensory parameters we included the ratio of soluble solids to titratable acidity (SSC/TA), since this ratio has been used to evaluate sweetness in some fresh produce (Magwaza and Opara, 2015). We found that sweetness was positively correlated with soluble solids, dry matter content and SSC/TA and negatively with ash content and titratable acidity. Fiber perception was positively correlated with ash content and negatively correlated with dry matter, soluble solids content and SSC/TA. Finally, the parameter off-flavors correlated positively with titratable acidity and ash content and negatively with the ratio SSC/TA.

**Table 5:** Correlations between chemical and sensory traits.

	Fiber	Off-flavors	Dry matter	Soluble solids content	pH	Titratable acidity	Ash	SSC/TA
Sweetness	−0.52^∗∗^	−0.74^∗∗∗^	0.47^∗∗^	0.52^∗∗^	−0.12	−0.38^∗^	−0.62^∗∗∗^	0.59^∗∗∗^
Fiber		0.28	−0.56^∗∗∗^	−0.58^∗∗∗^	−0.23	0.07	0.48^∗∗^	−0.43^∗^
Off-flavors			−0.21	−0.26	0.14	0.61^∗∗∗^	0.44^∗^	−0.60^∗∗∗^
Dry matter				0.97^∗∗∗^	−0.22	0.02	−0.87^∗∗∗^	0.61^∗∗∗^
Soluble solids content					−0.23	−0.01	−0.92^∗∗∗^	0.65^∗∗∗^
pH						0.12	0.35^∗^	−0.21
Titratable acidity							0.29	−0.75^∗∗∗^
Ash								−0.81^∗∗∗^

*SSC/TA: ratio of soluble solids to titratable acidity. ^∗^, ^∗∗^, ^∗∗∗^ indicates significant at P < 0.05, P < 0.01, and P < 0.001, respectively (Student’s t-test).*

## Discussion

The locations used for the experiment represented a wide range of variability on ‘calçot’ crop cultivation. In general, temperatures were the most variable parameters, especially between seasons. The differences in soil characteristics observed between locations can be attributed to the natural variation in soils throughout the territory and the differences in management practices among farmers (fertilization and soil tillage); these findings are representative of current ‘calçot’ production in Catalonia. Likewise, the varieties used in the experiment also are representative of current genotypic variability in farmers’ ‘calçot’ fields.

Scant research related with quality in ‘calçots’ has been published. Zudaire et al. (2017) determined the pH, soluble solids content, and titratable acidity in the juice of raw ‘calçot’; however, the methodological differences between their study and the current study make it difficult to compare results. Nevertheless, the orders of magnitude of the chemical parameters measured in our study were similar to those reported in roasted onions (Spanish food composition database, 2018) and raw onions (Barzegar et al., 2008; Petropoulos et al., 2015). Regarding the sensory analysis, the fact that the factor panelist was significant for all the traits considered indicates that panelists were applying the scales differently in their evaluations. The significance of the factor panelist is quite common in descriptive sensory analysis and it is related to slight differences in the reference values that judges learn (Romano et al., 2008). However, none of the interactions that included the panelist factor were significant, which means that, despite using different parts of the scale, the panel worked properly.

Our results show the important role of environmental factors in the chemical composition and sensory quality of ‘calçots.’ For all the chemical and sensory attributes studied, the effect of the environment was more important than the effect of the variety. Taking into account that our study is a first approximation, since no previous studies related with the influence of environmental variables or management practices have been done in ‘calçot’ cultivation, the results obtained will be helpful as a working basis for future research. The influence of environmental factors such as temperature, photoperiod, fertilization and/or other farming practices have been proved in the quality of crops (Hornick, 1992) such as onions (Sekara et al., 2017), tomatoes (*Solanum lycopersicum* L.) (Carli et al., 2011), globe artichoke (*Cynara scolymus* L.) (Lombardo et al., 2018) or leguminous vegetables (Ntatsi et al., 2018).

In our study, the role of genetic factors was less important. The chemical composition of ‘calçots’ from the four varieties studied was very similar, only differing in titratable acidity. Conversely, the factor variety was significant in sensory attributes. Sensory perception is highly complex, depending not only on chemical composition, but also on how volatile and non-volatile compounds interact, as has been studied in tomato (Baldwin et al., 2008). However, although the trained panel found statistically significant differences between the varieties for all the traits studied, these differences were limited in magnitude and would probably be undetectable for untrained consumers. These findings of low variability were to be expected as the four varieties included in this experiment are of the same varietal type and the improved varieties were derived from breeding the historical landrace. The principal difference between the varieties of ‘calçot’ used is the number of resprouts per plant. Breeding programs developed for ‘calçots’ have used the variability within the landrace. Roquerola, Montferri, and the new experimental variety (150,489; 164,668 and 175,656 ‘calçots’/ha respectively) were clearly more productive than the traditional population in this experiment (125,403 ‘calçots’/ha). Importantly, our results indicate that selection for increased production did not have a negative impact on quality, perhaps because the sensory profile was taken into account in these breeding programs to improve production (Simó et al., 2012a), though chemical composition was not controlled. Likewise, the present results are important because they show that the breeding program did not have an important impact on the chemical parameters studied; thus, it seems that a synchronic improvement of yield and quality-related traits may be possible for ‘calçots,’ in contrast with the dilution effect described for many other species (Morris and Sands, 2006).

The factor location was the main source of variation in the chemical and sensory parameters studied. The influence of growing site on quality parameters has been proved also in onions (Mallor et al., 2011; Lee et al., 2016) as well as other crops such as raspberries (*Rubus idaeus*) (Castilho Maro et al., 2014) or beans (*Phaseolus vulgaris* L.) (Florez et al., 2009). Our study included locations outside the area designated in the PGI; however, we found no clear pattern differentiating between the chemical composition of ‘calçots’ grown inside the PGI area and those grown outside this area. Nevertheless, with respect to the sensory profile, the values for the sensory attributes of ‘calçots’ grown in Viladecans (outside the PGI area) were the farthest from the ideotype. This approach should be further investigated, as it can improve the robustness of the quality label, as has been done in other products, such as beans (Florez et al., 2009), olive oil (Cosio et al., 2006), or wine (Díaz et al., 2003).

The time of year when ‘calçots’ were planted and harvested also had an important influence on the quality of ‘calçots.’ It is important to point out that differences in harvesting time did not only involve different environmental conditions. ‘Calçots’ of the late harvest usually had a slower development, so the differences found between harvests may also be due to some genetic differences. The present study has been useful in showing that these two factors had an influence on ‘calçots’ quality; however, due to the complexity of the experiment and the interactions between the environmental factors studied, more focused experiments must be done before solid recommendations can be given to farmers.

The experimental design of this study included different locations with a combination of soil properties and temperature effects, and different planting and harvesting times, which in the end, also represented different environmental options at a certain moment in the plant life cycle. The complexity of the environmental factors and their interactions provide us with an overview of the influence of the environment on ‘calçot’ crop, which had been never studied before. However, such a broad study makes it difficult to disentangle specific findings. From the two PCAs (**Figures 2**, **3**) and the direct correlations between chemical parameters and environmental data, we could infer that the chemical parameters studied could be explained through linear combinations of some of the conditions controlled in the experiment, but this relation is not easily described by practical equations. However, temperatures seemed to play an important role on the chemical parameters studied. Moreover, soil properties that could be modified through fertilization had an impact on the ‘calçots’; soil pH and sodium content influenced the pH of ‘calçots’ and, together with other cations, soil phosphorus content could influence the dry matter and soluble solids content of ‘calçots.’ The effect of both temperatures and fertilization in onion has been studied several times since these factors affect not only the plant development, but also the quality of the bulbs (Petropoulos et al., 2017; Sekara et al., 2017). The insights from this study allow us to speculate on future directions for research, but further studies will be required to grasp the complex factors underlying quality in ‘calçots.’

Trying to understand which environmental characteristics had an important influence on sensory attributes is even more complex, since only a subset of samples was analyzed. It is unfeasible to analyze a large number of samples via sensory analysis with trained panelists, because they can only assess a limited number of samples per testing session (Plans et al., 2014). Therefore, other approaches are necessary. Establishing relationships between chemical composition and sensory traits opens the door to approaches that can deal with large numbers of samples. Among the correlations between chemical and sensory parameters found in the present study, the significant positive correlation between sweetness and soluble solids content, a chemical parameter that has been widely used to indicate sweetness of fresh and processed horticultural products, seems especially promising (Magwaza and Opara, 2015). The correlation between sweetness and the ratio SSC/TA has been slightly higher but considering the increase of work on the analysis, the use of soluble solids content seems to be a better approach. Correlations between chemical parameters and sensory traits can be useful for breeding programs or quality control, where it may be necessary to work with large numbers of samples that would be impossible for panels to evaluate.

There are two possible approaches to improving nutritional composition or quality characteristics of ‘calçots’: breeding or modifying cultivation conditions. Since the variability among ‘calçots’ varieties is low, intravarietal variation must be exploited (Simó et al., 2013) and, if necessary, other varieties might be used to introduce new variability. Moreover, the negative estimated genotypic correlations between sweetness and the other two sensory attributes (fiber perception and off-flavors, both of which are undesirable) suggest that additional selection can bring ‘calçots’ closer to the sensory ideotype. However, our results show that much work remains to increase knowledge and improve crop management through factors such as irrigation and fertilization management, incidence of pests and diseases, effects of weather, type of soil, or weed management.

## Conclusion

The present study has generated new information regarding factors involved in the quality of ‘calçots,’ a crop barely investigated, enabling the influence of genetic and environmental factors in some key chemical and sensory traits of ‘calçots’ to be estimated.

Overall, the results point to a major environmental influence in the quality of ‘calçots’ cultivated from the most common varieties of BTL onion, including the original landrace. The low variability in the chemical composition and sensory traits among these varieties confirms that breeding programs to increase the production of ‘calçots’ plants have not significantly affected quality. Furthermore, this study has established correlations between sensory attributes and chemical parameters that can be useful when large numbers of samples need to be characterized in breeding or quality control. Finally, both breeding programs and crop management seem to be valid approaches to improving the commercial value of ‘calçots.’

## Author Contributions

SS performed the chemical analysis, conducted the testing sessions, participated in the interpretation of data and drafting the manuscript, gave final approval of the version to be published, and agreed to be accountable for all aspects of the work in ensuring that questions related to the accuracy or integrity of any part of the work are appropriately investigated and resolved. JC revised the article critically, gave final approval of the version to be published, and agreed to be accountable for all aspects of the work in ensuring that questions related to the accuracy or integrity of any part of the work are appropriately investigated and resolved. JS made substantial contributions to the conception or design of the work, participated in the analysis and interpretation of data, gave final approval of the version to be published, and agreed to be accountable for all aspects of the work in ensuring that questions related to the accuracy or integrity of any part of the work are appropriately investigated and resolved.

## Conflict of Interest Statement

The authors declare that the research was conducted in the absence of any commercial or financial relationships that could be construed as a potential conflict of interest.
